# Discovery and expression of insecticidal proteins via genome mining of novel *Bacillus thuringiensis* strain Bt1Fo

**DOI:** 10.3389/fmicb.2025.1679336

**Published:** 2025-10-24

**Authors:** Fazliddin B. Kobilov, Pengwei Li, Muhammad-Latif M. Nazirov, Meng Chen, Shakhlo Miralimova, Iskandar Yakubov, Akmal M. Asrorov, Khonsuluv Sohibnazarova, Nodira Azimova, Ilkhom Khalilov, Ulugbek Yusupov, Yihua Chen, Yue Tang, Zebinisa Mirakbarova

**Affiliations:** ^1^Institute of Microbiology, Uzbekistan Academy of Sciences, Tashkent, Uzbekistan; ^2^Research Institute of Plant Genetic Resources, Ministry of Agriculture, Tashkent, Uzbekistan; ^3^State Key Laboratory of Microbial Diversity and Innovative Utilization, Chinese Academy of Sciences, Beijing, China; ^4^Scientific Research Institute of Cotton Breeding, Seedling, and Growing Agrotechnologies, Tashkent, Uzbekistan; ^5^Faculty of Biology, National University of Uzbekistan, Tashkent, Uzbekistan; ^6^Institute of Bioorganic Chemistry, Uzbekistan Academy of Sciences, Tashkent, Uzbekistan; ^7^Alfraganus University, Tashkent, Uzbekistan; ^8^Center for Advanced Technologies, Tashkent, Uzbekistan; ^9^Institute of Fundamental and Applied Research, National Research University TIIAME, Tashkent, Uzbekistan; ^10^International Agriculture University, Tashkent, Uzbekistan; ^11^Institute of Biophysics and Biochemistry Under the National University of Uzbekistan, Tashkent, Uzbekistan; ^12^Central Asian University, Tashkent, Uzbekistan

**Keywords:** Bt1Fo, insecticidal proteins, Cry1Ia, genome mining, 3-domain, larval bioassay

## Abstract

The identification of environmentally friendly pesticides and insecticidal agents is crucial for developing sustainable agricultural practices. *Bacillus thuringiensis* exemplifies such a microbial agent, effectively controlling pests across a wide range of agronomic crops. In this study, we characterized the native strain Bt1Fo, which exhibits potent activity against major crop pests in the Lepidoptera order. All six types and truncated forms of the Cry1Aa and Cry1Ac insecticide proteins were expressed in *E. coli*, and recombinant proteins demonstrated strong activity against *Helicoverpa armigera*, with full-length Cry1Aa/1Ac and Vip3Aa being the most potent. Importantly, Cry1Ia exhibited high toxicity against *H. armigera*, representing its first documented efficacy against this major pest. These findings support Bt1Fo as a genetically stable source of multi-toxin biopesticides and provide a new opportunity for resistance management and further elucidation of the molecular interactions between insect midgut receptors and downstream signaling components.

## Introduction

Uzbekistan ranks among the top global cotton producers, with 1 million tons per year, and is currently the eighth-largest producer of cotton and the eleventh-largest exporter worldwide (USDA-FAS, 2025, April 9). The cotton bollworm (*H. armigera*), a major agricultural pest, inflicts significant damage on cotton crops across Uzbekistan, particularly in the Fergana, Andijan, Namangan, and Tashkent regions. Severe infestations were also documented in the Kashkadarya region's Nishon and Mirishkor districts in 2020 (Asrorov et al., 2020). Additionally, *H. armigera* harms other crops, including peas, eggplants, tomatoes, corn, and other legumes. Fighting against this pest using biotechnological approaches can lead to precise, sustainable, and environmentally friendly pest control strategies (Shang et al., 2024).

*B. thuringiensis* (Bt) is a Gram-positive, aerobic, spore-forming bacterium renowned for its entomopathogenic properties, primarily due to its production of insecticidal proteins. Among these, the parasporal crystalline δ-endotoxins, known as Cry proteins, are synthesized during the sporulation phase and exhibit specific toxicity against various insect orders, including *Lepidoptera, Coleoptera*, and *Diptera* (Jouzani et al., 2008). In addition to Cry proteins, *B. thuringiensis* produces other insecticidal proteins, such as Vip (vegetative insecticidal proteins), VipB (vegetative insecticidal proteins B), and Sip (secreted insecticidal proteins), during its vegetative growth phase (Domínguez-Arrizabalaga et al., 2020). The specificity and efficacy of these proteins have positioned *B. thuringiensis* as a pivotal agent in biological pest control strategies. Consequently, for several decades, extensive research has been dedicated to understanding and harnessing these toxins to manage agricultural pests effectively.

Bt-crops are genetically engineered crops that contain genes derived from the bacterium *B. thuringiensis*, enabling them to produce Cry proteins with insecticidal activity. These Bt plants, including cotton, corn, potatoes, soybeans, tomatoes, poplars, rice, and eggplants are widely used for effective pest control (Jouzani et al., 2017). Among these, the 3-domain Cry family is the most extensively studied, with numerous variants sharing a conserved structure and mode of action but differing in insect specificity (Bravo et al., 2013). According to Singh et al., transgenic cotton was developed using a truncated *cry1Ac* gene derived from *B. thuringiensis* subspecies *kurstaki* strain HD73. This construct result showed that single-copy insertion of *cry1Ac* gene can improve protein expression 2–2.5 times higher than Bt Cotton MON 531. MON 531 is a genetically engineered cotton variant of *Gossypium hirsutum* developed by Monsanto, marketed commercially as Bollgard™ Cotton (Singh et al., 2016). In addition to this, the pAY560325-cry1Ac vector construction successfully produced transgenic rice that harbors the 3-domain part of the *cry1Ac* gene, supporting the development of Agrobacterium-mediated transformation of rice varieties (Sri and Alifah, 2014).

As of 2025, the catalog of *B. thuringiensis* insecticidal genes has expanded significantly. Over 800 *cry* genes have been identified, classified into 75 distinct δ-endotoxin families, primarily located on large plasmids (Ragasruthi et al., 2024). Additionally, approximately 138 different *vip* genes, categorized into four groups (*Vip1*/*Vpb1, Vip2*/*Vpa2, Vip3*, and *Vip4*/*Vpb4*), have been identified and characterized (Jouzani et al., 2017; Gupta et al., 2021). Therefore, advanced sequencing technologies, including next-generation sequencing (NGS) and Nanopore sequencing, are being employed to identify novel *B. thuringiensis* toxins and a diverse array of insecticidal genes.

Despite the broad utility of Bt-based products, the evolution of resistance among target pests and the limited scope of existing Cry and Vip toxins underscore a critical need for novel, locally adapted insecticidal agents. In other studies, whole-genome mining of Bt strains (SY49.1) has led to the discovery of previously unknown insecticide variants and bioactive compounds with enhanced pesticidal and antimicrobial properties (Yilmaz et al., 2025). In another study, approximately 3,000 Bt isolates were screened using a high-throughput assay to identify Cry proteins effective against *Helicoverpa zea*. After filtering out already-known Cry1Ac and Cry2A variants, PCR-based sequencing of 3-domain from 48 active isolates revealed novel *cry1B*-type 3-domain sequences, including one associated with a *cry1Bj* variant and another linked to orphan open reading frames, suggesting natural 3-domain swapping via horizontal recombination as a generator of novel toxins (Cong et al., 2024). Another line of research involved whole-genome sequencing and bioinformatic mining of diverse Bt strains. In a Turkish Bt isolate, researchers identified multiple *cry* gene fragments, including a previously unreported *Cry2Aa18* variant, along with other bioactive sequences. These genomic explorations reinforce the potential for mining Bt biodiversity to uncover structurally novel insecticidal proteins useful for microbial control and next-generation engineering strategies (Aswathi et al., 2024). By coupling whole-genome sequencing with proteomic LC-MS/MS, this study identified two entirely new Bt toxin candidates (Peg5936 and Peg5937) with less than 36% similarity to known toxins. Mining via combined “omics” approaches can thus uncover structurally novel proteins with potential for next-generation Bt traits (Khorramnejad et al., 2020). These discoveries underscore the continuous efforts to expand the repertoire of *B. thuringiensis* toxins for enhanced pest control strategies.

In this study, we report the complete genome sequencing and annotation of the native Bt1Fo strain, isolated from a dead *Galleria mellonella* larva in the Tashkent region of Uzbekistan. Genome analysis and PCR results of Bt1Fo revealed six insecticidal genes (*cry1Aa, cry1Ac, cry1Ia, cry2Ab, cry2Ab*, and *vip3Aa*). Recombinant proteins expressed in *E. coli* exhibited potent activity against *H. armigera* larvae in bioassays. These findings suggest that Bt1Fo strain and its insecticidal derivatives have significant potential for managing lepidopteran pests and for developing Bt-based transgenic crops tailored to agricultural conditions in Uzbekistan.

## Materials and methods

### Bacterial strains, plasmids, and culture conditions

The local Bt1Fo was isolated from the Lepidopteran family species *G. mellonella* in 2014 and collected in the Molecular Biology Department of the Institute of Microbiology, Uzbekistan Academy of Sciences. The culture of *B. thuringiensis* strain Bt1Fo was grown in Luria-Bertani (LB) medium at 30 °C for 16 h with vigorous shaking, or on LB agar plates under the same temperature conditions. Bt1Fo was characterized by cultural and morphological signs using an NLCD-307B-2 light microscope (magnification: 400 × ). The plasmids and bacterial strains used in this research are given in Supplementary Table 1. Colonies resistant to antibiotics were selected on LB plates supplemented with 50 μg/mL of kanamycin. General DNA cloning was performed using *E. coli* JM109. For protein expression, *E. coli* BL21 (DE3) was used. Strains of *E. coli* were grown at 37 °C using LB broth and agar. During the protein expression process, a temperature of 16 °C was used for *E. coli* strains. The plasmid pET-28a (+) and pSEVA234 were protein expression vectors. The T7 promoter (pET-28a) and the TRC promoter (pSEVA234) were induced for expression of the recombinant insecticide genes using 0.2 mM IPTG. Then, induction was performed over 18 h under 16 °C, after which positive colonies were selected for protein extraction.

### gDNA isolation and genomic sequencing

Bt1Fo was grown in LB media, and total gDNA was isolated as described by a modified version of the Marmur method (Salvà-Serra et al., 2018). The extracted gDNA was measured using a Nanodrop (NanoPhotometer N60; IMPLEN), and its integrity was evaluated by gel electrophoresis (0.8% agarose). The genome sequencing of Bt1Fo was conducted using the Nextera XT DNA Library preparation kit on an Illumina HiSeq 2500 platform, employing a paired-end 2 × 150 bp approach.

### Genome and plasmid assembly

The quality of the Illumina short reads was assessed and controlled using FastQC (v0.11.8) (Wingett and Andrews, 2018). Sequencing reads were trimmed with Cutadapt v4.4 to remove adapters, low-quality bases (Phred < 30), ambiguous nucleotides, and reads < 50 bp (Martin, 2011). DNA assembly was performed using SPAdes version 4.0.0 in “careful” mode, using paired-end trimmed reads as input. Six threads were allocated for computation, and k-mer sizes were set to 21, 33, 55, and 77 (16 and. Assembled contigs were scaffolded into four scaffolds using Multi-CSAR (Liu et al., 2022). The quality of contigs and scaffolds was evaluated using QUAST v5.3.0 (Supplementary Figure 1; Gurevich et al., 2013), while the assembly completeness was assessed with BUSCO v5.5.0 (Supplementary Figure 2; Simão et al., 2015). The assembly graphs were visualized via Bandage v0.9.0 (Wick et al., 2015). Plasmid sequences were retrieved from WGS reads and reconstructed using PlasmidSPAdes (Antipov et al., 2016). Classification of assembled reads was performed using Kraken2 (Wood et al., 2019).

### Functional annotation and genome analyses

Functional annotation and in-depth characterization of scaffolded genomes and plasmids were performed using Bakta (Software: v1.8.2, Database: v5.0, light) and The RAST Server: Rapid Annotations using Subsystems Technology (Aziz et al., 2008; Overbeek et al., 2014; Brettin et al., 2015). Genomic islands and horizontally acquired genes were predicted using Islandviewer (Bertelli et al., 2017). Annotation of prophage regions and classification of viral signals were performed using Phigaro (Starikova et al., 2020) and VirSorter (29) with the following input parameters: min_score = 0.5, min_length = 500.

### Insecticide gene identification

We initially planned to identify the insecticide genes from Bt1Fo strain. Based on the primary PCR result for detecting *cry1A, cry2A*, and *vip* gene families, and RAST results, primers for *cry1Aa, cry1Ab, cry1Ac, cry1Ia, cry2Aa, cry1Ab*, and *vip3Aa* genes (Supplementary Table 2) were designed via SnapGene software and synthesized by Azenta Life Sciences (Beijing, China). Due to the high sequence similarity observed in the third domain of the truncated *cry1Aa, cry1Ab*, and *cry1Ac* genes, as revealed by alignment analyses, distinguishing among these genes necessitated the design of specific primers and the implementation of a two-step PCR strategy.

All the insecticidal toxic genes were amplified from the genomic DNA of Bt1Fo strain. The 50 μL PCR reaction contained 100 ng DNA template, 25 μL of 2 × Phanta buffer, 1 μL of each dNTP, 0.3 μL Phanta HiFi (Labs, Vazyme), 2 μl of up and down primers, and double-distilled water up to a final volume of 50 μL. The PCR protocol consisted of an initial denaturation at 94 °C for 5 min, followed by 35 cycles of denaturation at 94 °C for 15 s, primer annealing at 58 °C for 15 s, and extension at 72 °C for 2 min. A final extension was performed at 72 °C for 10 min.

### Insecticide gene cloning

In order to construct the expression plasmids of insecticidal toxic genes from Bt1Fo strain, we amplified the coding sequences of several target genes, purified, and then inserted into the *Nde*I/*Xho*I sites of pET28a and the *Kpn*I/*Pst*I sites of pSEVA234. For the construction of these plasmids, the linear pET28a fragment was amplified from the free gene pET28a plasmid with pET28a-*Xho*I **F** and pET28a-*Nde*I **R** primers, and pSEVA234 vector was restricted with *Kpn*I/*Pst*I enzymes. After cloning, vectors harboring the target genes were transferred into the cloning *E. coli* JM109 strain using the heat-shock transformation method. Five colonies from each gene transformant were picked for PCR confirmation. The plasmid DNA was extracted from the positive colonies, and the target plasmids were confirmed by DNA sequencing (Azenta Life Sciences).

After sequencing, the up- and down-reads of the genes were assembled. Then, they were compared and blasted to the NCBI BLAST database. All the sequenced insecticidal toxic genes showed 100% similarity with *cry1Aa, cry1Ac, cry1Ia, cry2Aa, cry2Ab*, and *vip3Aa*, respectively. The nucleotide sequence of the genes, deposited to NCBI database, were given the accession number *cry1Aa* (PP897827), *cry1Ac* (PP897828), *cry1Ia* (PP897829), *cry2Aa* (PP907159), *cry2Ab* (PP907160), *vip3Aa* (PP897826) and the proteins were named as Cry1Aa, Cry1Ac, Cry1Ia, Cry2Aa, Cry2Ab, Vip3Aa by the *B. thuringiensis* Toxin Nomenclature Committee.

### Phylogeny

The 16S rRNA was proposed as a molecular marker to differentiate *Bacillus cereus* and *B. thuringiensis*. However, studies have demonstrated that these genes exhibit high sequence similarity between the two species, making discrimination challenging (Chen and Tsen, 2002). The sequences of the 16S rRNA and insecticide resistance genes were analyzed using the standard nucleotide-nucleotide BLAST and provided to the GeneBank database. Sequence alignment was performed using MEGA11 software, and a phylogenetic tree was constructed by the maximum-likelihood (ML) method to infer evolutionary relationships among the taxa. The robustness of the tree was assessed through 1,000 bootstrap replicates, resulting in a consensus tree that reflects the evolutionary history of the analyzed taxa (Saitou and Nei, 1987).

### Protein expression and purification

The target plasmids were transferred into *E. coli* BL21 (DE3) and all the insecticidal toxic proteins were expressed. A single transformant colony of *E. coli* BL21, which harbors a specific protein expression plasmid, was grown in a liquid LB medium containing 50 μg/mL kanamycin. Then, the samples were cultured for 16 h at 37 °C, 220 rpm. An overnight bacterial culture was diluted 1:1,000 into the LB medium containing 50 μg/mL kanamycin and incubated at 37 °C with shaking at 220 rpm until the optical density at 600 nm (OD600) reached 0.6. Protein expression was then induced by adding IPTG to a final concentration of 0.2 mM, and the culture was incubated at 16 °C with shaking at 180 rpm for 18 h.

Protein purification was further carried out as described in our previous work (Li et al., 2021). Protein purification was conducted at 4 °C using a Ni-NTA affinity column, following the manufacturer's guidelines. After the cell pellets were resuspended in a binding buffer (20 mM Tris-HCl, 500 mM NaCl, 5 mM imidazole, pH 7.9) for ultrasonication. After sonication, cell debris was removed by centrifugation, and the resulting supernatant was applied to a pre-equilibrated Ni-NTA affinity column. The column was washed with washing buffer (20 mM Tris-HCl, 500 mM NaCl, 60 mM imidazole, pH 7.9), and the bound proteins were eluted using a 20 mM Tris-HCl, 500 mM NaCl, 500 mM imidazole, pH 7.9 solution. The eluted fractions containing the target protein were pooled, desalted using PD-10 columns (GE Healthcare, USA), and concentrated via ultracentrifugation with an Amicon Ultra Centrifugal Filter device (Merck Millipore, USA, molecular mass cutoff of 10 kDa). The purified proteins were mixed with 10% glycerol and stored at −80 °C. Protein concentrations were determined using the Bradford assay (Bradford, 1976).

### Protein analysis by SDS-PAGE

SDS-PAGE was conducted to separate insecticidal proteins expressed in *E. coli* BL21, following the method described by Laemmli (1970). Protein samples were prepared by mixing with SDS sample buffer and heating at 95 °C for 10 min, and electrophoresed on 12% resolving and a 5% stacking polyacrylamide gels using a Mini-PROTEIN system (Bio-Rad). Gels were stained with Coomassie R-250 and destained using a standard acetic acid/ethanol solution. Protein molecular weights were estimated using pre-stained protein standards (Bio-Rad).

### Western blotting

Western blotting of proteins was performed following the manufacturer's instructions. For this purpose, purified insecticidal proteins were transferred onto a PVDF membrane (Millipore) using a semi-dry blotting system (Bio-Rad). The membrane was blocked with 1% BSA in TBS and incubated with anti-His-Tag antibody (1:1,000) for 1 h at 25 °C. The membrane was washed with TBS-T solution. Then, the membrane was incubated for 1 h in TBS supplemented with universal anti-mouse antibodies (1:10,000). After the washing step, the membrane was visualized with DAB reagent.

### Larval rearing for bioassay

Cotton bollworm (*H. armigera*) larvae were collected from a field cleared of cucumbers in the Kuyi-Chirchik district and reared under controlled laboratory conditions. The larvae were fed fresh alfalfa (*Medicago sativa*) leaves daily until they pupated. Emerging male and female moths (3–4 pairs per jar) were placed in glass jars maintained at 26 °C, 75% relative humidity, with a photoperiod of 10 h light / 14 h dark, and were provided with a 10 % sucrose solution as nourishment. Eggs were laid within 3 days, and larvae hatched 2 days later. Neonate larvae were fed on tender sorrel and alfalfa leaves. Upon reaching the second instar, larvae were individually placed in Petri dishes containing filter paper for subsequent bioassays (Sekhar Dash et al., 2020).

### Insect bioassay and statistical analysis

Purified Cry and Vip3Aa proteins were evaluated for their efficacy against second-instar *H. armigera* larvae. For each treatment, 20 larvae were individually placed in separate Petri dishes (one larva per dish) with four replicates per treatment (*n* = 80 larvae per protein). A working solution containing 500 ng of total protein was applied to the fresh alfalfa leaf surface (4.5 cm^2^). This corresponds to a nominal applied dose of 112 ng/cm^2^ for most proteins. Based on SDS-PAGE densitometry, the effective doses were estimated to be 72 ng/cm^2^ for Cry1Aa and 30 ng/cm^2^ for Cry1Ac. Leaves treated with deionized water served as the negative control. Larval mortality was recorded daily for four consecutive days. Mortality data from the 20 individually housed larvae per replicate were used to calculate mean mortality rates and standard deviations (SD) at each time point using OriginPro software. The resulting data were analyzed using one-way ANOVA to compare mortality among treatment groups at *p* ≤ 0.05.

## Results

### A potential strain selection

**Figure 1:**
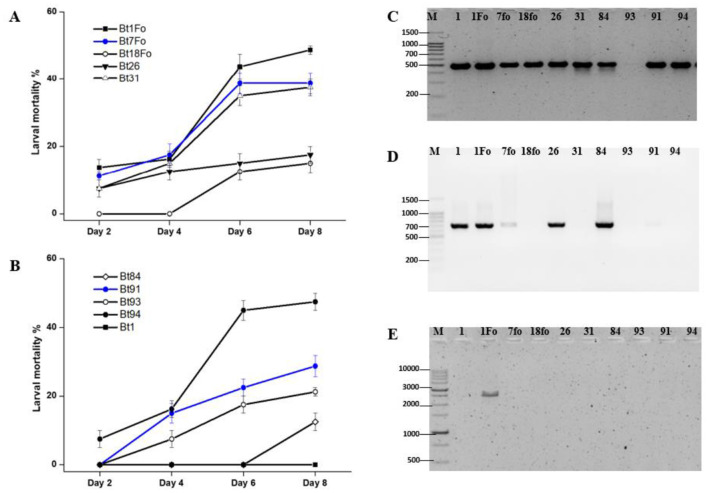
Larval mortality and gene amplification profiles of *B. thuringiensis* isolates. **(A, B)** Larval mortality (%) of different Bt isolates against target 4–to 5-day-old *H. armigera* larvae over 8 days. Mortality data are presented as mean ± SE from four independent replicates (*n* = 40). Statistical differences among treatments at each time point were evaluated using one-way ANOVA (*p* < 0.05). PCR results of the *cry1A* **(C**-490 bp), the *cry2A* **(D**-701 bp), and the *vip3Aa* **(E**-2,370 bp) genes in the tested *B. thuringiensis* strains. Gene-specific primer sequences are listed in Supplementary Table 2.

In order to identify a potent *B. thuringiensis* strain among local isolates, bioassays and PCR analyses were conducted targeting insecticidal genes, including the *cry1A* and *cry2A* gene families, as well as the *vip3Aa* gene. An eight-day bioassay was conducted to evaluate the efficacy of various *B. thuringiensis* strains against 4–to 5-day-old *H. armigera* larvae. The observed larval mortality rates were as follows: Bt1 – 0%, Bt1Fo – 50%, Bt7Fo – 40%, Bt18 – 15%, Bt26 – 18%, Bt31 – 40%, Bt84 – 13%, Bt91 – 30%, Bt93 – 20%, and Bt94 – 50%. PCR analysis revealed that nine out of 10 *B. thuringiensis* strains possessed the *cry1A* gene, except for Bt93. The *cry2A* gene was detected in strains Bt1, Bt1Fo, Bt7Fo, Bt26, and Bt84, but was absent in Bt18, Bt31, Bt93, Bt91, and Bt94. Notably, only Bt1Fo strain harbored the *vip3Aa* gene among the 10 strains analyzed. These results indicate that strains Bt1Fo and Bt94 exhibited the highest larval mortality, suggesting strong insecticidal activity. Based on the bioassay results, both Bt1Fo and Bt94 strains exhibited 50% mortality against the target lepidopteran pest. However, molecular characterization revealed that Bt1Fo harbors a broader spectrum of lepidopteran-active genes, including *cry1A, cry2A*, and notably *vip3Aa*, compared to Bt94. Given the presence of these additional insecticidal genes, particularly *vip3Aa*, which has demonstrated high efficacy against various lepidopteran pests, Bt1Fo was selected for further investigation (Figure 1).

### Morphological characterization

**Figure 2:**
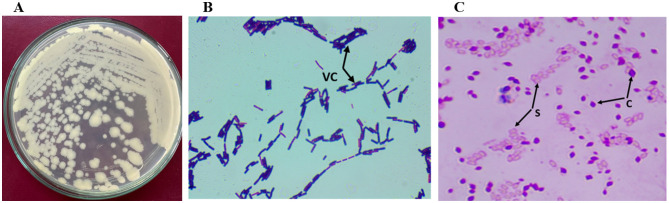
Morphological characteristics of Bt1Fo strain. **(A)** Colony morphology after 48 h of growth on LB agar; **(B)** vegetative cells' view under the light microscope (VC); **(C)** Light microscopic view of spores (S), and parasporal crystals **(C)**. Magnification 1,000 × **(B, C)**.

The color of Bt1Fo colonies is cream. The growth of the strain is slightly raised to convex with a somewhat elevated center, spreading with some rounded areas; the reverse side is cream-colored. The colony is easily removed using a bacterial loop, exhibiting a stretchy but non-slimy texture. Diameter: 0.5–0.8 μm at 1 day; endospores formed on the 4th day, measuring 0.4 μm, 0.5 μm, and 0.6 μm. One-day-old cells are motile (Figure 2).

### Genome characterization

The Bt1Fo strain genome comprised a 5,981,074 bp circular chromosome. The genome assembly benchmarking run in *prok_genome* mode demonstrated high completeness, with 100% of BUSCOs identified using the bacteria_odb10 dataset, comprising 95.2% single-copy and 4.8% duplicated BUSCOs, indicating a high-quality assembly with no fragmented or missing genes. The assembly consists of 4 scaffolds and 246 contigs, with a total length of 5.98 Mb, 0.405% gaps, a scaffold N50 of 5 Mb, and a contig N50 of 85 kb. The assembly was further evaluated using QUAST, which confirmed that it comprised four contigs, with the largest contig measuring 5,491,109 bp. Each plasmid assembly resulted in one contig. The chromosome assembly had a GC content of 34.80%, an N50 value of 5,491,109 bp, and an L50 of 1. The Kraken2-based taxonomic classification of the assembled genome revealed that all sequencing reads were confidently assigned to *B. thuringiensis*, with a 100% accuracy.

**Figure 3:**
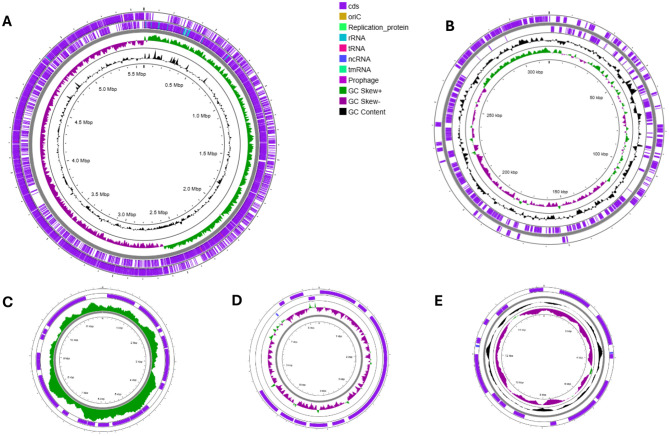
Schematic representation of Bt1Fo genome: circular map of chromosome **(A)** and plasmids pBt1Fo (**B)**, PBT_comp3 **(C)**, PBT_comp4 **(D)**, PBT_comp5 **(E)**. The scale is shown in megabases (Mbp) on the inside circle. GC content is represented in black, and GC skew (G-C)/(G+C) in violet and green. The purple-striped circles represent the reverse and forward strands of DNA, including coding sequences, tRNA, ncRNA, rRNA, tmRNA regions, replication origin sites, and gapped regions (visualized in colors according to the legend).

Visualization of the assembled and annotated chromosome revealed an asymmetric distribution of GC base pairs across the genome. In contrast, the plasmids displayed a more symmetric GC base composition, highlighting the high heterogeneity of bacterial chromosome structure (Figure 3).

Functional annotation in Bakta identified a total of 6,494 genomic features, including 6,002 CDSs, 112 tRNAs, 3 rRNAs, 19 ncRNAs, 107 ncRNA regions, 2 CRISPR loci, 4 sORFs, 1 tmRNA, 2 oriC sites, and 242 gaps (Table 1). Among these, 541 genes were annotated as hypothetical proteins.

**Table 1:** Genomic features of Bt1Fo strain short read assembly: chromosome and plasmids (Bakta Report).

**Replicon name**	**Length (bp)**	**Contigs**	**GC content (%)**	**Genes**	**CDS**	**ncRNA**	**tRNA**	**rRNA**	**tmRNA**
Chromosome Bt1Fo	5,491,109	4	34.8	6,331	6,002	126	112	3	1
pBt1Fo	317,339	1	33.2	333	294	–	–	–	–
PBT_comp3	11,846	1	31.8	27	24	–	–	–	–
PBT_comp4	8,317	1	29.7	14	11	1	–	–	–
PBT_comp5	15,865	1	31.6	29	19	2	–	–	–

Table 1 illustrates the genomic features of Bt1Fo strain, as reported by Bakta. It includes features from the chromosome assembly and four plasmids: pBt1Fo, PBT_comp3, PBT_comp4, and PBT_comp5. The table shows the distribution of coding sequences (CDS), non-coding RNAs (ncRNA), tRNAs, rRNAs, tmRNAs, the origin of replication (oriC), small open reading frames (sORFs) and gapped sites.

**Figure 4:**
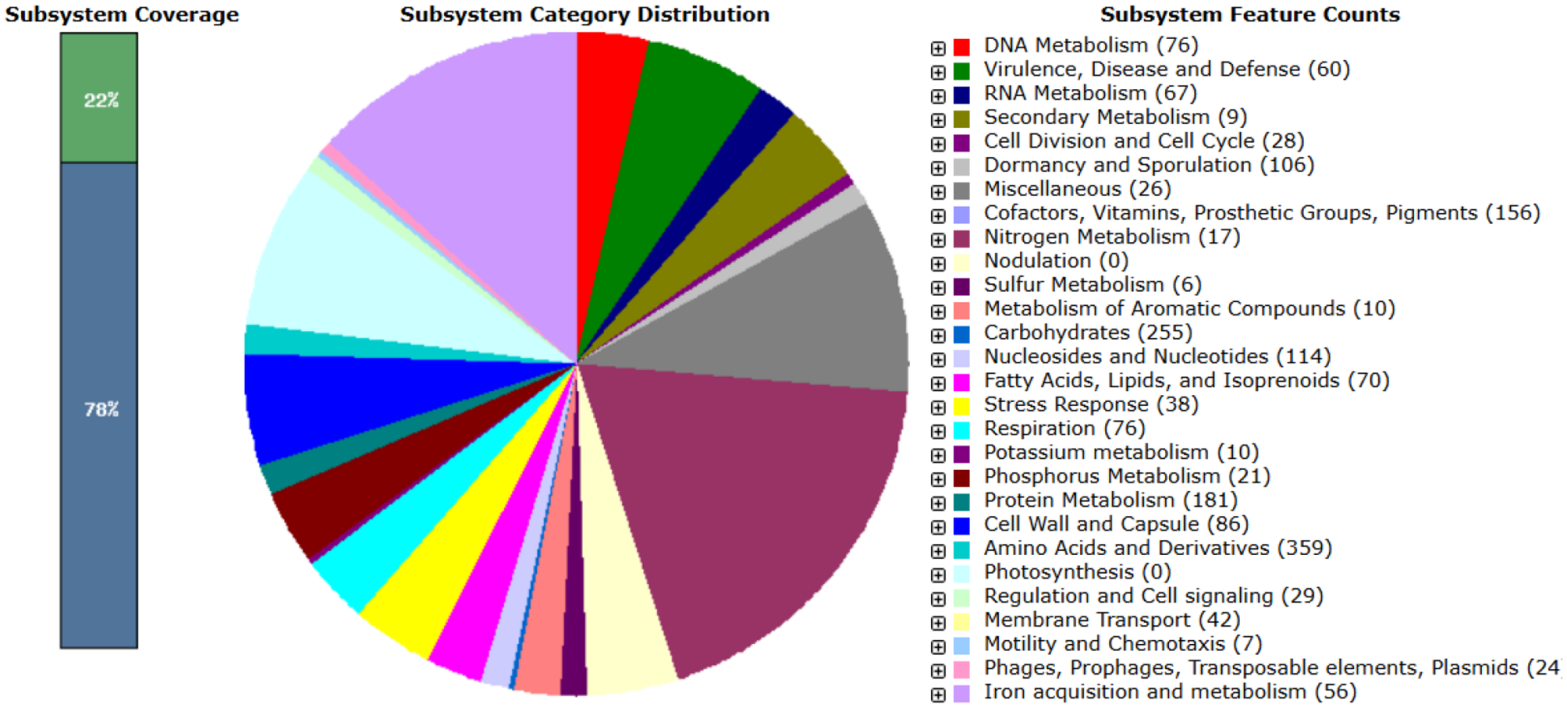
The summary of subsystem categories associated with the genome of Bt1Fo. A pie chart illustrates the number of features within each subsystem and their respective coverage.

Further functional annotation of Bt1Fo genome, as predicted by the RAST server, identified 126 ncRNAs, comprising 112 tRNAs, 3 rRNAs, and 1 tmRNA. A total of 6,331 coding sequences were assigned to 339 subsystems, with major functional categories involving amino acid metabolism (359 features), protein metabolism (181), carbohydrate metabolism (255), cofactor and vitamin biosynthesis (156), and stress response (38). Categorized all genes into 28 subsystems, according to their role in metabolic functions (Figure 4).

Resistome analysis of Bt1Fo genome using RGI (CARD) revealed multiple antibiotic resistance genes, including *vanY, vanT*, and *vanW* across several vancomycin clusters (*vanA, vanB, vanG, vanI, vanF, vanM*), as well as *tetB(P)* for tetracycline, *FosB* for fosfomycin, and β-lactamase genes (*BcI, BcIII*), indicating β-lactam resistance. Additionally, the absence of the virulence genes *hbl*A, *hbl*C, *hbl*D, *nhe*A, *nhe*C, and *cap*A was confirmed by genomic analysis.

Throughout the analysis, we identified 60 genes associated with virulence, disease, and defense mechanisms, including genes related to resistance to antibiotics and toxic compounds (46), and genes involved in invasion and intracellular resistance (14). Moreover, we identified 20 phage-related elements, including genes encoding phage capsid proteins, phage packaging machinery, phage replication functions, phage tail fiber proteins, and phage tail proteins, with four genes in each category. Plasmid annotation identified the insecticidal crystal protein genes *cry2Aa* (1.9 kb), *cry2Ab* (1.9 kb), *cry1Ia* (2.2 kb), *cry1Ac* (3.5 kb), and *cry1Aa* (3.5 kb) on pBt1Fo plasmid. Further BLASTn analysis revealed the highest sequence similarities of these Cry proteins to those of *B. thuringiensis* strain CP158700.1. pBt1Fo prophage analysis identified five prophage-like regions (1,604–36,493 bp), grouped into four clusters. Most prophages were linked to the Siphoviridae family, except prophage_3 (Myoviridae) and two prophage helix-turn-helix proteins were identified outside predicted genomic islands.

A total of 28 genomic islands (GIs) were identified in Bt1Fo genome, including 3 plasmid-borne and 25 chromosomal. Chromosomal islands ranged from 3.6 kb to 48.2 kb and were distributed across multiple loci. None of the chromosomal islands contained cry genes, indicating that insecticidal functions in Bt1Fo are plasmid-encoded. Several of these islands contained phage proteins, with at least one cluster embedded within a predicted island. Three GIs identified on the plasmid of Bt1Fo: 45,209–55,459 bp (10,250 bp) containing a phage-related protein, 103,042–113,956 bp (10,914 bp) harboring cryB1 and cry2Aa, and 150,178–160,295 bp (10,117 bp) with no insecticidal genes. Notably, cry2Ab (130,524–132,425 bp) lies outside predicted GIs, ~17 kb downstream of the second GI. In addition, cry1Ia (116,483–118,642 bp, - strand) and cry1Aa (119,149–122,679 bp, - strand) are positioned between xerS (115,024–116,268 bp, + strand) and the autolysin-encoding gene xlyA (122,969–123,925 bp, - strand), but not within a GI. Similarly, *cry1Ac* (70,337–73,870 bp, - strand) is located upstream of xlyA (74,160–75,116 bp, - strand), again outside predicted islands. Analysis of transposable elements of Bt1Fo identified 14 transposase encoding loci, from which DDE transposase was located ~2.4 kb upstream of *cry1Ac* in plasmid pBt1Fo, followed by an additional transposase (~7.7kb downstream). Similarly, the *cryB1*–*cry2Aa* cluster was located downstream of a Tn3-family transposase (~10.5kb). Two HTH-OrfB-IS605 domain containing proteins were identified in pBt1Fo plasmid downstream of *cry2Ab* (~1.7 kb) and right upstream of DDE transposase (~105 bp), preceding *cry1Ac*.

**Figure 5:**
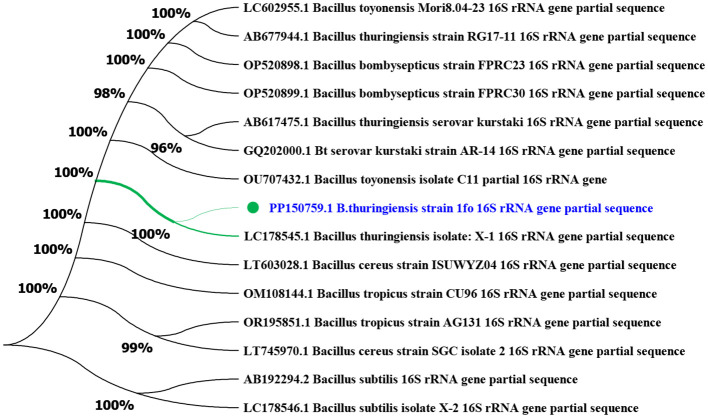
Phylogenetic tree of Bt1Fo strain based on the 16S rRNA gene (MEGA-X, Maximum Likelihood method, 1,000 bootstrap replications). Bt1Fo strain compared to a total of 15 *Bacillus* strains by the 16S rRNA gene.

Using the maximum likelihood statistical method in MEGA11 bioinformatics software, phylogenetic trees of Bt1Fo strain were constructed based on its 16S rRNA gene sequence. The Bt1Fo strain was compared with *B. toyonensis, B. bombysepticus, B. cereus, B. subtilis*, and *B. thuringiensis* based on its 16S rRNA gene (Figure 5).

Phylogenetic analysis based on 16S rRNA gene sequences places Bt1Fo strain (PP150759, highlighted in blue) firmly within *B. thuringiensis* lineage, showing 100% bootstrap support (Figure 5). This result confirms its taxonomic placement within the *B. thuringiensis* lineage. The high sequence similarity to Bt1Fo and other reference strains suggests a conserved evolutionary background, aligning with previous studies on the taxonomy of *B. thuringiensis*. Despite the overall conservation, minor sequence variations may indicate strain-specific adaptations. We selected *B. toyonensis* and *B. bombysepticus* for phylogenetic contrast, as both species are evolutionarily close to *B. thuringiensis* within the *B. cereus* group. Importantly, *B. toyonensis* shares Cry/Cyt-type insecticidal genes characteristic of Bt strains and showed high activity against the Lepidopteran *Cydia pomonella*, the Coleopteran *Anthonomus grandis*, and low activity against the Dipteran *Aedes aegypti*. Meanwhile, *B. bombysepticus* produces a novel parasporal crystal toxin with documented pathogenicity toward silkworms and Cry1Ac-resistant *H. armigera* strains (Sauka et al., 2022; Lin et al., 2015).

### Primary and secondary PCR screening for insecticide genes

**Figure 6:**
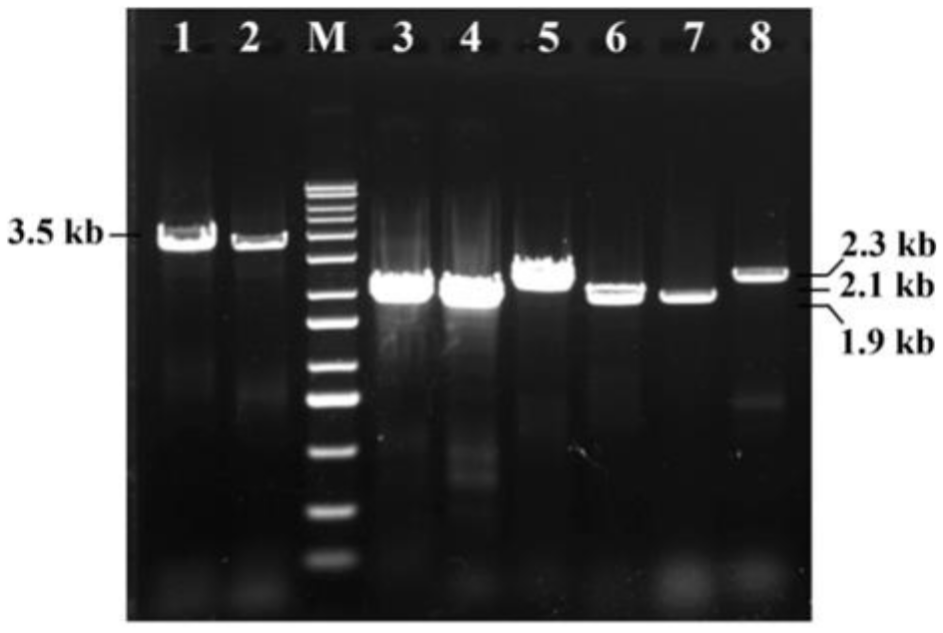
Six types of insecticide genes PCR results of Bt1Fo strain. M-1kb DNA ladder. 1–*cry1Aa* full-length (3.5 kb); 2–*cry1Ac* full-length (3.5 kb); 3–*cry1Aa* 3-domain variant (1.9 kb); 4–*cry1Ac* 3-domain variant (1.9 kb); 5–*cry1Ia* gene (2.1 kb); 6–*cry2Aa* (1.9 kb); 7–*cry2Ab* (1.9 kb); 8–*vip3Aa* (2.3 kb).

Initial PCR screening of Bt1Fo strain using universal primers targeting the *cry1, cry2*, and *vip3Aa* gene families successfully amplified these genes, indicating the presence of multiple insecticidal toxin genes within Bt1Fo genome. After that, the secondary PCR result showed Bt1Fo possessed specific Cry Gene Subtypes. Subsequent secondary PCR analyses using gene-specific primers identified the presence of the *cry1Aa, cry1Ac, cry2Aa*, and *cry2Ab* genes, but did not detect the *cry1Ab* gene. Detecting these insecticide genes suggests a broad-spectrum insecticidal potential, as Cry1 and Cry2 proteins are known for their activity against lepidopteran pests, while Vip3Aa protein extends the insecticidal range to other orders. The coexistence of these specific *cry* gene subtypes within a single strain is noteworthy, as it may enhance the strain's efficacy against a broader range of insect pests. Whole-genome sequencing followed by Rapid Annotations using Subsystems Technology (RAST) annotation confirmed the presence of the *cry1Ia* gene in Bt1Fo. Notably, the *cry1Ia* gene encodes a Cry protein variant known for its unique insecticidal properties, including toxicity against both lepidopteran and coleopteran larvae (Song et al., 2003). The identity of all *cry1Aa, cry1Ac, cry1Ia, cry2Aa, cry2Ab*, and *vip3Aa* (Figure 6) adds to the strain's potential as a versatile biocontrol agent.

**Figure 7:**
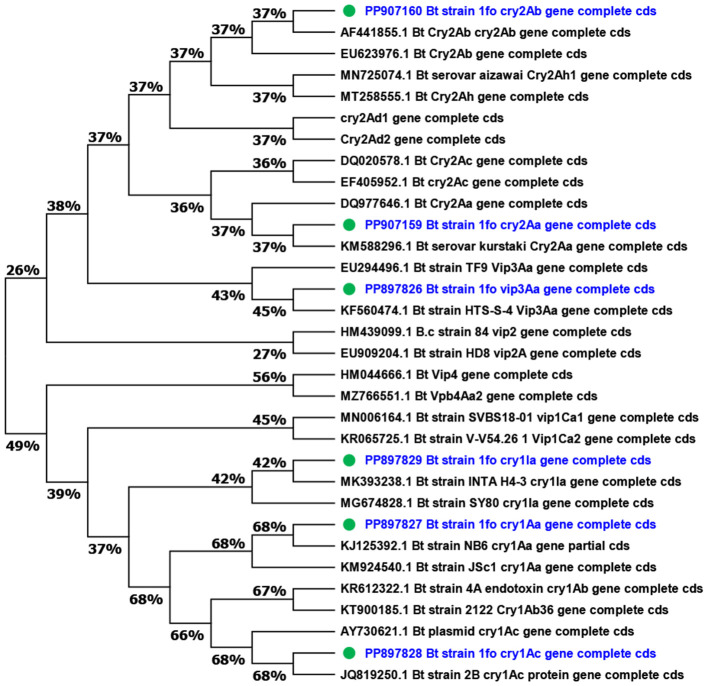
Phylogenetic analysis of insecticide genes of Bt1Fo strain. The bioinformatic tool MEGA 11 was used to create a phylogenetic tree of the *cry* and *vip* genes based on multiple sequence alignments using Cluster W. To quantify the support for each branch, the trees were built using the maximum joining (MJ) approach with 1,000 bootstrap replicates. The blue-marked genes belonged to Bt1Fo (PP150759.1).

In order to construct phylogenetic relationships of the insecticidal toxic genes, the MEGA 11 software was used. The phylogenetic tree consisted of *cry1, cry2*, and *vip* family genes which include *cry1Aa, cry1Ab, cry1Ac, cry1Ia, cry2Aa, cry2Ab, cry2Ac, cry2Ad, cry2Ah, vip1Ca, vip2A, vip3Aa*, and *vip4Aa* family genes were used (Figure 7).

### SDS-PAGE and western blot analysis

**Figure 8:**
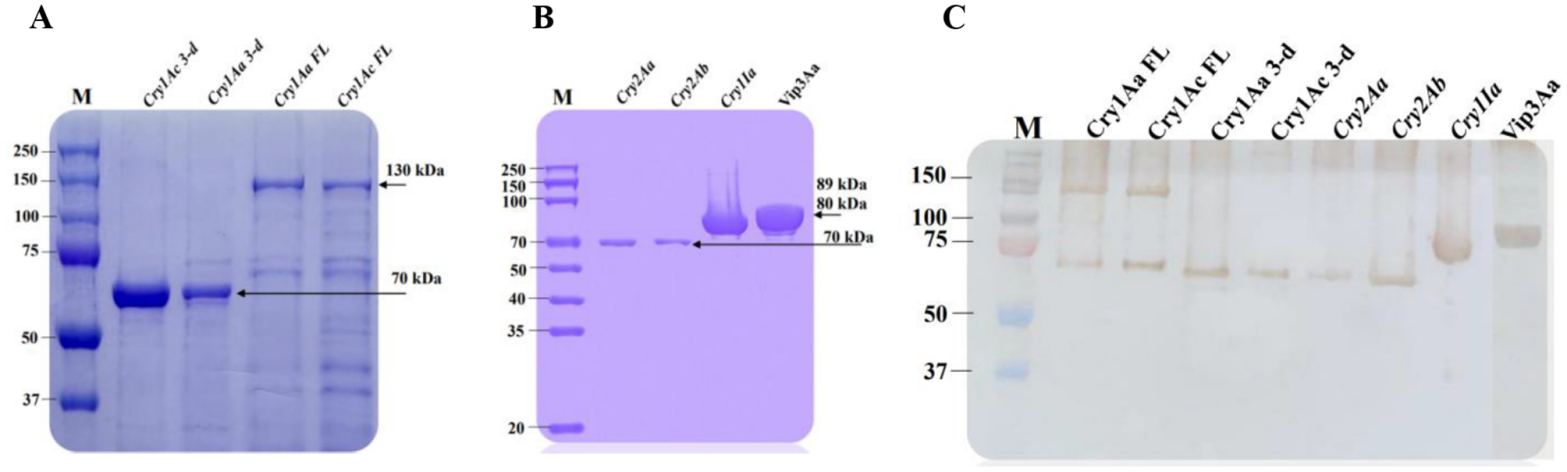
SDS-PAGE and Western blot analysis of insecticide proteins of Bt1Fo strain. M-protein ladder. **(A)** line 1-Cry1Ac 3-domain; line 2-Cry1Aa 3-domain; line 3-Cry1Aa full-length; line 4-Cry1Ac full-length. **(B)** line 1-Cry2Aa; line 2-Cry2Ab; line 3-Cry1Ia; line 4-Vip3Aa. **(C)** line 1-Cry1Aa full-length; line 2-Cry1Ac full-length; line 3-Cry1Aa 3-domain; line 4-Cry1Ac 3-domain; line 5-cry2Aa; line 6-Cry2Ab; line 7-Cry1Ia; line 8-Vip3Aa.

All amplified insecticide genes were cloned into the pET-28a (+) and pSEVA234 vectors for protein expression using *E. coli* BL21 expression strain. After expression and purification, the extracted proteins were analyzed and compared using SDS–PAGE and Western blotting. As illustrated in Figure 8, a clean recombinant insecticide protein band of 130 kDa (Cry1Aa and Cry1Ac full-length), 89 kDa (Vip3Aa), 80 kDa (Cry1Ia), 70 kDa (Cry2Aa and Cry2Ab), and 70 kDa (Cry1Aa and Cry1Ac 3-domain) were detected. These findings indicate that all insecticidal protein-coding sequences were successfully induced, producing sufficient quantities for subsequent analyses, including western blotting and bioassays.

To confirm the identity and molecular weights of the expressed insecticidal proteins, western blot analysis was conducted on the purified proteins from the previously mentioned colonies. Using mono-specific anti-His antibodies, distinct immunoreactive bands were detected at approximately 130 kDa, 89 kDa, 80 kDa, and 70 kDa, corresponding to the expected sizes of the target proteins. These results validate the successful expression and integrity of the His-tagged insecticidal proteins, as visualized in Figure 8.

### Insect bioassays

**Figure 9:**
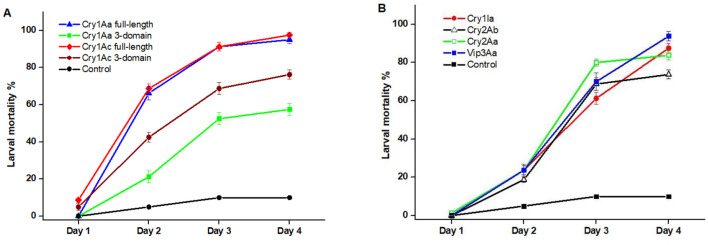
Larval mortality induced by recombinant Bt1Fo toxins over four days. **(A)** Mortality rates (%) of larvae treated with Cry1Aa full-length, Cry1Aa 3-domain, Cry1Ac full-length, and Cry1Ac 3-domain proteins compared to the control. **(B)** Mortality rates (%) of larvae exposed to Cry1Ia, Cry2Ab, Cry2Aa, and Vip3Aa proteins compared to the control. Control groups were treated with water without the toxin. 112 ng/cm^2^ protein concentrations was applied in all treatments. Data represent mean ± SE from four independent replicates (*n* = 80). One-way ANOVA revealed significant differences in mortality among treatments at each time point, with full-length proteins significantly more effective than their truncated forms (*p* < 0.05).

The insecticidal efficacy of purified proteins Cry1Aa (full-length and 3-domain variant), Cry1Ac (full-length and 3-domain variant), Cry1Ia, Cry2Aa, Cry2Ab, and Vip3Aa was evaluated against neonate larvae of *H. armigera*. The full-length Cry1Aa and Cry1Ac proteins exhibited the highest toxicity, achieving nearly 100% mortality, whereas their truncated three-domain variants were less potent with 60% and 80%, respectively. This indicates that the C-terminal domains contribute more importantly to toxicity and stability. Cry1Ac achieved comparable or greater larval mortality at less than half the effective dose, underscoring its superior potency. The mortality rates for Cry1Ia, Cry2Aa, Cry2Ab, and Vip3Aa almost reached 90%, 80%, 70%, and 90%, respectively. The control groups exhibited a baseline mortality rate of 10%. These findings underscore the potent insecticidal activity of full-length Cry1Aa and Cry1Ac against *H. armigera*, suggesting that the 3-domain variants, along with other tested proteins, also hold promise for effective pest control strategies (Figure 9).

## Discussion

To our knowledge, Cry1Ia's toxicity against *H. armigera* has not previously been reported, and a novel finding of this study is that Cry1Ia caused approximately 90% mortality in *H. armigera* larvae. In previous studies, Cry1Ia was demonstrated to have high activity against lepidopteran *Ostrinia nubilalis* (Khorramnejad et al., 2022), and Cry1Ia7 protoxin also showed toxicity against *E. insulana, L. botrana*, and *P. xylostella* and the coleopteran *L. decemlineata*, but displayed no toxicity to *B. mori, H. armigera, M. sexta, S. exigua, S. frugiperda, S. littoralis*, and *T. ni*, even at a high dose of 100 μg/ml (Ruiz De Escudero et al., 2006). Additionally, Martínez et al. (2005) found Cry1Ia appeared among *B. thuringiensis* isolates toxic toward *H. armigera* in spore-crystal form, but did not test with a purified Cry1Ia protein alone. Thus, our work represents the first demonstration that purified Cry1Ia is highly toxic to *H. armigera*. By contrast, our bioassays demonstrate to our knowledge that *H. armigera* is also susceptible to purified Cry1Ia protein. This expands the known host range of Cry1Ia and underscores its potential utility in controlling cotton bollworm. The result suggests that Cry1Ia could be incorporated into Bt crop pyramids targeting multiple pests. Future work should investigate whether Cry1Ia binds to any of the canonical *H. armigera* midgut cadherin or aminopeptidase receptors differently from Cry1A or Cry2 toxins, as cross-resistance with Vip3Aa has been noted only in limited cases (Bachler et al., 2025).

Notably, both protoxin and truncated variants Cry1Ac consistently exhibited higher toxicity against *H. armigera* larvae compared to Cry1Aa. Although the nominal applied dose was identical (112 ng/cm^2^) for most proteins, densitometry revealed effective doses of 72 ng/cm^2^ for Cry1Aa and only 30 ng/cm^2^ for Cry1Ac. Thus, Cry1Ac achieved comparable or superior larval mortality at less than half the effective dose, confirming its higher intrinsic potency. Structural and functional properties explain this difference. Cry1Ac binds with greater affinity to multiple midgut receptors including cadherin, APN, and ALP than Cry1Aa and possesses more versatile Domain II loops that enhance receptor interactions (De Maagd et al., 2001; Pigott and Ellar, 2007; Pacheco et al., 2009). In addition, Cry1Ac protoxin exhibits greater stability against gut protease degradation, ensuring that more active toxin reaches its targets (Hernández-Rodríguez et al., 2013).

Domain III of the three-domain Cry proteins is known to contribute to proper folding, receptor binding, and membrane insertion (Thammasittirong et al., 2019). For instance, the Cry4Ba toxin's C-terminal fragment binds insect midgut membranes and is proposed to anchor the toxin to lipid bilayers. By analogy, the superior activity of full-length Cry1A compared to DIII truncated variants suggests that removing the C-terminal domains impairs structural stability or receptor interaction. Collectively, our results highlight both the necessity of maintaining the full three-domain architecture for maximal efficacy and the superior intrinsic potency of Cry1Ac over Cry1Aa in killing *H. armigera* larvae.

The Bt1Fo strain's suite of toxins offers complementary modes of action. Cry2Aa and Cry2Ab (80% and 70% mortality) act via receptors distinct from those of Cry1 toxins, as Cry1 and Cry2 families are not cross-resistant in most pests. Our Vip3Aa (90%) adds further diversity, and Vip3 proteins bind different gut targets than Cry proteins, a fact that is exploited in commercial Bt cotton containing Vip3Aa in combination with Cry1Ac/Cry2Ab (Bachler et al., 2025). Together, Cry1, Cry2, and Vip3Aa toxins can be combined in pyramided biopesticides to delay resistance. Notably, receptor-binding studies show Vip3A interactions are usually distinct from Cry1/Cry2, with negligible cross-resistance. This mosaic of targets means a pest resistant to one toxin remains vulnerable to others. In addition, previous studies have reported a potential synergistic interaction among Cry1, Cry2, and Vip3Aa against *Spodoptera frugiperda* larvae (Soares Figueiredo et al., 2019). Although we did not experimentally assess such interactions, exploring potential synergy under our assay conditions represents a valuable direction for future research. Moreover, the presence of all three insecticidal protein classes in a single, genetically stable strain like Bt1Fo makes it a promising platform for developing pyramided transgenic crops or formulated biopesticide products.

In a genomic context, we analyzed the Bt1Fo genome for the distribution of insecticidal genes. Coverage patterns and plasmid scaffolding revealed plasmid-borne cry loci. Specifically, Cry1Ia, Cry1Aa, and Cry2Ab are positioned on the plasmid outside predicted genomic islands, flanked by recombinase (xerS) and autolysin (xlyA) genes, which may act as recombination hotspots. In contrast, CryB1 and Cry2Aa were located within a plasmid genomic island that also contained phage proteins, consistent with horizontal gene transfer. This organization suggests that plasmids not only encode the primary insecticidal determinants of Bt1Fo but also provide genomic plasticity through mobile elements.

Recent CRISPR/Cas9 studies highlight how key receptors like cadherin (HaCad) mediate Cry1Ac toxicity, knocking out HaCad in *H. armigera* confers >500-fold Cry1Ac resistance (Wang et al., 2016). Such knowledge informs future work that we propose using ligand-binding assays or CRISPR knockout of candidate receptors like cadherin, APNs, and ABC transporters to pinpoint how Bt1Fo Cry1, Cry2, and Vip3 toxins interact with *H. armigera*. Computational docking and structural modeling could also predict binding interfaces for Cry1Ia and Vip3Aa on novel receptors.

Bt1Fo represents a unique and potent biocontrol strain, and broad-spectrum toxins offer both biotechnological advantages and ecological safety for pest control. Some extensive meta-analyses and field studies demonstrate that Bt strains and Bt-crops typically have minimal adverse effects on non-target arthropod communities compared to conventional chemical insecticides (Yu et al., 2011). Moreover, many beneficial species, such as predators and parasitoids, are often more abundant in Bt-treated environments. Cry proteins generally undergo rapid degradation in soil or are adsorbed onto organic matter, limiting environmental persistence and reducing the likelihood of broad ecological exposure (Helassa and Staunton, 2013). Importantly, Bt1Fo lacks the virulence genes *hbl*A, C, D, *nhe*A, C, and *cap*A. Previous studies have shown that the *nhe*ABC and *hbl*CDA operons are widespread in the *B. cereus* sensu lato group, where their existence increases the risk of gastrointestinal toxicity (Böhm et al., 2015), thereby further supporting Bt1Fo's suitability as a safe biocontrol agent.

Overall, the local Bt1Ffo strain represents a genetically stable and ecologically safe microbial agent. Future studies should focus on determining binding targets for these proteins and assessing their combined efficacy in the field to inform next-generation biopesticide design.

## Data Availability

The genome of Bacillus thuringiensis 1Fo has been deposited in NCBI GenBank under accession numbers CP195215 and CP195218, Bioproject accession number PRJNA1282158, and BioSample number SAMN49630233. Additional supplemental data are available in the Supplementary material accompanying this article.
