# Supplementary material for: Discovery and expression of insecticidal proteins via genome mining of novel Bacillus thuringiensis strain Bt1Fo
Source: Front Microbiol. 2025 Oct 24;16:1679336. doi: 10.3389/fmicb.2025.1679336 (PMC12593462; doi:10.3389/fmicb.2025.1679336)
Supplement: Supplementary file 1 [file Supplementary_file_1.docx]

**Supplementary Table 1. Bacterial strains and plasmids.**

| **Strains and plasmids** | **Characteristics*** | **Reference or source** |
| --- | --- | --- |
| ***Bacillus thuringiensis* 1Fo** | Used identity and amplification of the insecticide genes | This work |
| ***Escherichia coli*** |  |  |
| JM109 | General cloning | Invitrogen |
| BL21 (DE3) | Host for protein expression | Novagen |
| **Plasmids** |  |  |
| pET28a | Kan^r^, protein production vector | Novagen |
| pSEVA234 | Kan^r^, protein production vector | SEVA |
| pSEVA234::Cry1Aa-3 domain | Kan^r^, for producing Cry1Aa 3 domain | This work |
| pET28a::Cry1Aa-full length | Kan^r^, for producing Cry1Aa full-length | This work |
| pET28a::Cry1Ac-full length | Kan^r^, for producing Cry1Ac full-length | This work |
| pET28a::Cry1Ac-3 domain | Kan^r^, for producing Cry1Ac 3 domain | This work |
| pET28a::Cry1Ia | Kan^r^, for producing Cry1Ia | This work |
| pET28a::Cry2Aa | Kan^r^, for producing Cry2Aa | This work |
| pET28a::Cry2Ab | Kan^r^, for producing Cry2Ab | This work |
| pET28a::Vip3Aa | Kan^r^, for producing Vip3Aa | This work |

Kan^r^, kanamycin resistance.

**Supplementary Table 2. Primers used in this study**

| **Primers** | **Sequence (5′ to 3′)*** | **Uses** |
| --- | --- | --- |
| cry1A-F  cry1Aa-R | **GGTACC**aaaggagatatacatatggataacaatccgaacatc  **CTGCAG**tcagtggtggtggtggtggtggga | Amplification of the *cry1Aa* 3 domain part |
| cry1A-F cry1Ac-R | taagaaggagatata**CATATG**gataacaatccgaacatc  tggtgatggtggtg**CTCGAG**agtaactggaataaattc | Amplification of the *cry1Ac* 3 domain part |
| cry1A-F cry1Aa-R | gtgccgcgcggcagc**CATATG**gataacaatccgaacatc ggtggtggtggtggtg**CTCGAG**ctattcctccataagaagta | Amplification of the *cry1Aa* full-length part |
| cry1A-F cry1Ac-R | gtgccgcgcggcagc**CATATG**gataacaatccgaacatc ggtggtggtggtggtg**CTCGAG**ctattcctccataaggag | Amplification of the *cry1Ac* full-length part |
| cry1Ia-F cry1Ia-R | ctggtgccgcgcggcagc**CATATG**aaactaaagaatcaag ggtggtggtggtggtg**CTCGAG**ctacatgttacgctcaatatg | Amplification of the *cry1Ia* |
| cry2Aa-F cry2Aa-R | ctggtgccgcgcggcagc**CATATG**aataatgtattgaatag tggtggtggtggtg**CTCGAG**ttaataaagtggtggaagattag | Amplification of the *cry2Aa* |
| cry2Ab-F cry2Ab-R | ctggtgccgcgcggcagc**CATATG**aatagtgtattgaatag tggtggtggtggtg**CTCGAG**ttaataaagtggtgaaatattag | Amplification of the *cry2Ab* |
| vip3Aa-F vip3Aa-R | ctggtgccgcgcggcagc**CATATG**aacaagaataatactaaat tggtggtggtggtg**CTCGAG**ttacttaatagagacatcg | Amplification of the *vip3Aa* |
| **The designed restriction site in each primer is capitalized and underlined* | | |
| **Primers used to differentiate the 3 domain parts of the *cry1Aa*, *cry1Ab*, and *cry1Ac* genes** | | |
| cry1A-unvr-F cry1Aa-R | atggataacaatccgaacatc  tcatccagacaaaattcatct | First amplification of the cry1Aa 3 domain part |
| cry1A-unvr-F cry1Ab-R | atggataacaatccgaacatc  ggcacattttccgattg | First amplification of the cry1Ab 3 domain part |
| cry1A-unvr-F cry1Ac-R1 | atggataacaatccgaacatc  ggttgcctattaatgtctttg | First amplification of the cry1Ac 3 domain part |
| cry1A-unvr-F  cry1A(a/b)-R | atggataacaatccgaacatc  atattctgcctcaaaggtta | Second amplification of the ry1Aa/1Ab 3 domain part |
| cry1A-unvr-F  cry1Ac-R2 | atggataacaatccgaacatc  atattcagcctcgagtgttg | Second amplification of the cry1Ac 3 domain part |
| **Primers used to amplify the *cry1A*, *cry2A*, and vip3Aa genes from local Bt strains** | | |
| Lep1A  Lep1B | ccggtgctggatttgtgtta  aatcccgtattgtaccagcg | cry1Aa, cry1Ab, cry1Ac  (490 bp). |
| cry2F  cry2R | gttattcttaatgcagatgaatggg  cggataaaataatctgggaaatagt | cry2  (701 bp) |
| vip3AF  vip3AR | atgaacaagaataatactaaa  gcggccgcttacttaatagagac | vip3A (2370 bp) |

**Insecticide Gene Identification**

We initially planned to identify the insecticide genes from Bt1Fo strain. Based on the primary PCR result for detecting *cry1A*, *cry2A*, and *vip* gene families, and RAST results, primers for *cry1Aa*, *cry1Ab*, *cry1Ac*, *cry1Ia*, *cry2Aa*, *cry1Ab*, and *vip3Aa* genes (Supplementary Table 2) were designed via SnapGene software and synthesized by Azenta Life Sciences (Beijing, China). Due to the high sequence similarity observed in the third domain of the truncated *cry1Aa*, *cry1Ab*, and *cry1Ac* genes, as revealed by alignment analyses, distinguishing among these genes necessitated the design of specific primers and the implementation of a two-step PCR strategy. A universal forward primer, cry1A-unvr-F, was designed based on the highly conserved *N*-terminal regions of *cry1Aa*, *cry1Ab*, and *cry1Ac*, and utilized for the amplification of all three genes. In order to achieve gene-specific amplification in the first PCR step, reverse primers cry1Aa-R, cry1Ab-R, and cry1Ac-R1 were individually paired with cry1A-unvr-F to selectively amplify cry1Aa, cry1Ab, and cry1Ac, respectively. Following successful gene separation, a second PCR step was conducted: for *cry1Aa* and *cry1Ab*, cry1A-unvr-F was paired with cry1A (a, b)-R; for cry1Ac, cry1A-unvr-F was paired with cry1Ac-R1 (Supplementary Table 2). This two-step PCR approach enabled the specific amplification and differentiation of the closely related *cry1A* gene variants.

All the insecticidal toxic genes were amplified from the genomic DNA of Bt1Fo strain. The 50 µL PCR reaction contained 100 ng DNA template, 25 µL of 2× Phanta buffer, 1 µL of each dNTP, 0.3 µL Phanta HiFi (Labs, Vazyme), 2 μl of up and down primers, and double‑distilled water up to a final volume of 50 µL. The PCR protocol consisted of an initial denaturation at 94°C for 5 minutes, followed by 35 cycles of denaturation at 94°C for 15 seconds, primer annealing at 58°C for 15 seconds, and extension at 72°C for 2 minutes. A final extension was performed at 72°C for 10 minutes.

**Schematic representation of the primers to differentiate the 3 domain parts of the *cry1Aa*, *cry1Ab*, and *cry1Ac* genes**


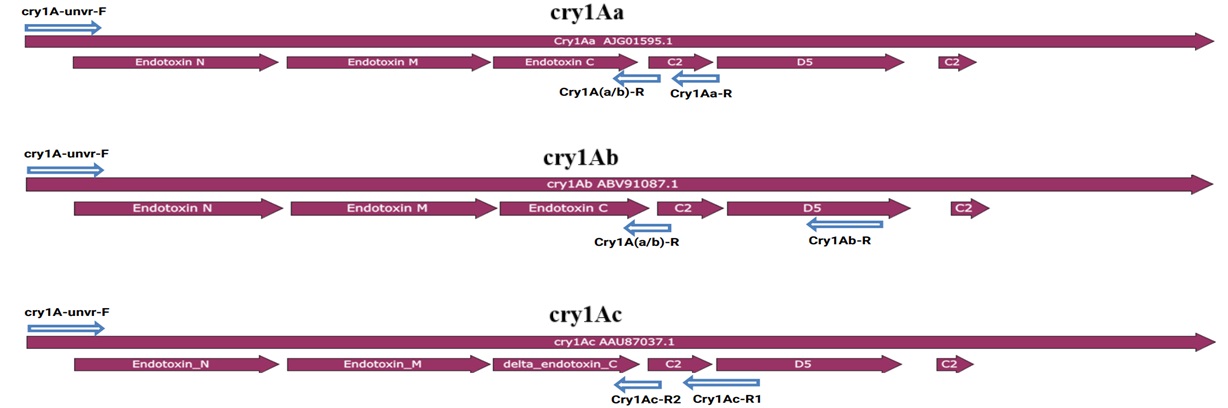


**Insecticide Gene Cloning**

In order to construct the expression plasmids of insecticidal toxic genes from Bt1Fo strain, the 3.5-kb *cry1Aa*, 1.9-kb *cry1Aa*, the 3.5-kb *cry1Ac*, 1.9-kb *cry1Ac*, the 2.1-kb *cry1Ia*, the 1.9-kb *cry2Aa* gene, the 1.9-kb *cry2Ab*, and the 2.3-kb *vip3Aa* were amplified, purified, and inserted into the *Nde*I/*Xho*I sites of pET28a to get the plasmids pET28a-cry1Aa, pET28a-cry1Ac, pET28a-cry1Ia, pET28a-cry2Aa, pET28a-cry2Ab, pET28a-vip3Aa and the *Kpn*I/*Pst*I sites of pSEVA234 to get the plasmids pSEVA234-cry1Aa (3-domain). For the construction of these plasmids, the linear pET28a fragment was amplified from the free gene pET28a plasmid with pET28a-*Xho*I **F** and pET28a-*Nde*I **R** primers, and pSEVA234 vector was restricted with *Kpn*I/*Pst*I enzymes. They were then purified from agarose gel by FastPure Gel DNA Extraction Mini Kit, following the manufacturer's recommended methodology. The homologous sequences found in each of the 8 genes allowed for ligation using the linear pET28a fragment using a one-step cloning technique. After cloning, vectors harboring the target genes were transferred into the cloning *E. coli* JM109 strain using the heat-shock transformation method. Five colonies from each gene transformant were picked for PCR confirmation. The plasmid DNA was extracted from the positive colonies, and the target plasmids were confirmed by DNA sequencing (Azenta Life Sciences).
